# Corticotropin‐releasing hormone–mediated blood pressure elevation during acute stress in male rats

**DOI:** 10.14814/phy2.71172

**Published:** 2026-11-13

**Authors:** Si Liang, Yu Tian, Ran Li, Jia Wen Li, Jia Xin Li, Hui Zhuo Jia, Si Yun Niu, Hong Jie Wang, Na Li

**Affiliations:** ^1^ Department of Anesthesiology Affiliated Hospital of Hebei University Baoding China; ^2^ Clinical Medical College, Hebei University Baoding China; ^3^ Basic Medical College, Hebei University Baoding China; ^4^ Hebei Key Laboratory of Aging and Health Hebei University Baoding Hebei China

**Keywords:** acute stress, blood pressure, cardiovascular, corticosterone, corticotropin‐releasing hormone (CRH), hypothalamus, hypothalamus‐pituitary‐adrenal gland (HPA) axis

## Abstract

Acute stress triggers autonomic responses, characterized by increases in arterial blood pressure and sympathetic nerve activity, and a neuroendocrine response, characterized by an increase in corticosterone level. However, the mechanisms underlying elevation of blood pressure during stress remain unclear. In this study, we investigated the link between neuroendocrine neurons releasing corticotropin‐releasing hormone (CRH) and elevation of blood pressure during acute restraint stress. In Sprague–Dawley rats, restraint stress increased blood pressure. Selected time points during restraint showed increased serum corticosterone (CORT) and norepinephrine (NE) concentrations, consistent with activation of the HPA axis and sympathetic nerve activity. CRH knockdown in the PVN significantly blunted the rise in blood pressure during restraint stress and post‐restraint CORT and NE levels. In addition, pretreatment with the CRHR1 antagonist antalarmin attenuated the restraint‐induced HR response and reduced post‐restraint CORT and NE levels. Blood pressure responses were numerically smaller after antalarmin, but the treatment × stress interactions for SBP, DBP, and MAP were not significant. In summary, these data suggested that CRH signaling mediates stress‐induced blood pressure responses through coordination of the HPA axis and sympathetic nervous system. Our findings highlight the CRH–CRHR1 axis as a potential target for preventing cardiovascular complications associated with acute stress.

## 1 INTRODUCTION

Hypertension is a leading risk factor for cardiovascular morbidity and mortality. According to the World Health Organization, an estimated 1.4 billion adults aged 30–79 years worldwide were living with hypertension in 2024 (Organization WH, 2025). Among its diverse etiological factors, psychological stress has been increasingly recognized as a key contributor to hypertension pathogenesis (Brotman et al., 2007; Ulrich‐Lai & Herman, 2009). Previous studies and reviews indicate that chronic psychosocial stress is relevant to hypertension; epidemiological evidence includes workplace strain and anxiety‐related disorders, while neurobiological work has characterized ascending pathways that integrate stress‐related autonomic and endocrine responses (Landsbergis et al., 2013; Lim et al., 2021; Myers et al., 2017). Moreover, stress‐induced increases in blood pressure are a strong predictor of future hypertension independent of baseline resting blood pressure (Carroll et al., 2012), emphasizing the clinical importance of understanding acute stress responses. Stress can be broadly defined as a real or perceived threat to homeostasis or well‐being that elicits coordinated neural, autonomic, and neuroendocrine responses (Myers et al., 2017). Acute and chronic stress differ in duration and physiological consequences, with repeated or prolonged exposure promoting persistent cardiovascular and neuroendocrine adaptations (Brotman et al., 2007; Ulrich‐Lai & Herman, 2009).

Stress profoundly activates both the locus coeruleus–norepinephrine (LC–NE) system and the two major stress axes—the sympathetic–adrenal–medullary (SAM) axis and the hypothalamic–pituitary–adrenal (HPA) axis. Activation of the SAM axis results in rapid increases in heart rate, vascular resistance, and blood pressure through catecholamine release, whereas activation of the HPA axis induces broader metabolic, cardiovascular, and immune effects through glucocorticoid secretion (Ulrich‐Lai & Herman, 2009). Within the HPA axis, the paraventricular nucleus (PVN) of the hypothalamus serves as a central regulatory hub. Parvocellular PVN neurons secrete corticotropin‐releasing hormone (CRH) in response to stress, integrating excitatory inputs from limbic and brainstem centers. CRH release is critical for corticosterone (CORT) secretion and the orchestration of stress‐related behavioral and physiological adaptations (Fuzesi et al., 2016; Sukhareva, 2021). Acute stress enhances the activity of PVN‐CRH neurons, whereas repeated exposure to homotypic stressors leads to progressive habituation of CRH‐neuron responses (Kim et al., 2019). Importantly, although direct evidence of PVN‐CRH alterations in hypertensive patients remains limited, experimental studies in animal models, particularly SHR, have demonstrated enhanced PVN‐CRH signaling activity, increased CRH mRNA and protein expression, and heightened PVN neuronal activity (Qin et al., 2018). These findings suggest a potential role of PVN‐CRH dysregulation in hypertension development (Zhang et al., 2023).

Although acute stress‐induced blood pressure arises from the interplay of central and peripheral mechanisms—including PVN‐CRH neuron activation, HPA axis stimulation, and sympathetic drive—the precise contribution of CRH/HPA axis signaling to rapid stress‐related blood pressure elevation remains unclear. A major unresolved question is that if CRH exerts pressor effects predominantly through endocrine glucocorticoid‐dependent pathways or if CRH acts directly within central autonomic circuits to drive sympathetic outflow. Understanding this distinction is crucial, as conventional antihypertensive strategies primarily target peripheral vascular rather than central stress integration. This present study investigates the mechanistic relationships among CRH, CORT, NE, and acute stress‐induced blood pressure elevation. Understanding these interactions may inform early‐stage prevention efforts and the development of novel therapeutic targets for stress‐related cardiovascular disease. Clarifying the contribution of PVN‐CRH signaling will help to identify upstream neural drivers of stress‐induced sympathetic hyperactivation and support new neuromodulatory or pharmacological interventions aimed at reducing stress reactivity and preventing the transition from transient to chronic hypertension.

## 2 MATERIALS AND METHODS

### 2.1 Animal and ethical approval

Male SD rats weighing 200–250 g were sourced from SPF (Beijing Biotechnology Co., Ltd.,) and housed at Hebei University Animal Experimental Center. The rats were maintained in groups of 4 to 5 per cage, with ad libitum access to standard laboratory rat chow (Changsheng Biotechnology, China; Cat. No. D001) and water, and bedding was changed biweekly. The animal facility was kept at 20°C–25°C with 40%–70% relative humidity and a 12‐h light/dark cycle, adhering to specific pathogen‐free (SPF) standards. Following a 1‐week acclimation period, the rats were enrolled in the study. All procedures were approved by the Hebei University Institutional Review Board (Approval No. HBU2024R024).

### 2.2 Experimental grouping

The experiment consisted of three stages: (1) Establishment of an acute stress model through restraint in 10 rats (*n* = 10). (2) Acute stress experiment following CRH knockout using the CRISPR/Cas9 technique. The rats were divided into a knockdown (KD) group and a control (CON) group. (3) Assessment of acute stress following CRHR1 intervention. Based on specific experimental requirements, the rats were further divided into an antalarmin group and a vehicle control group (Figure 1).

**FIGURE 1:**
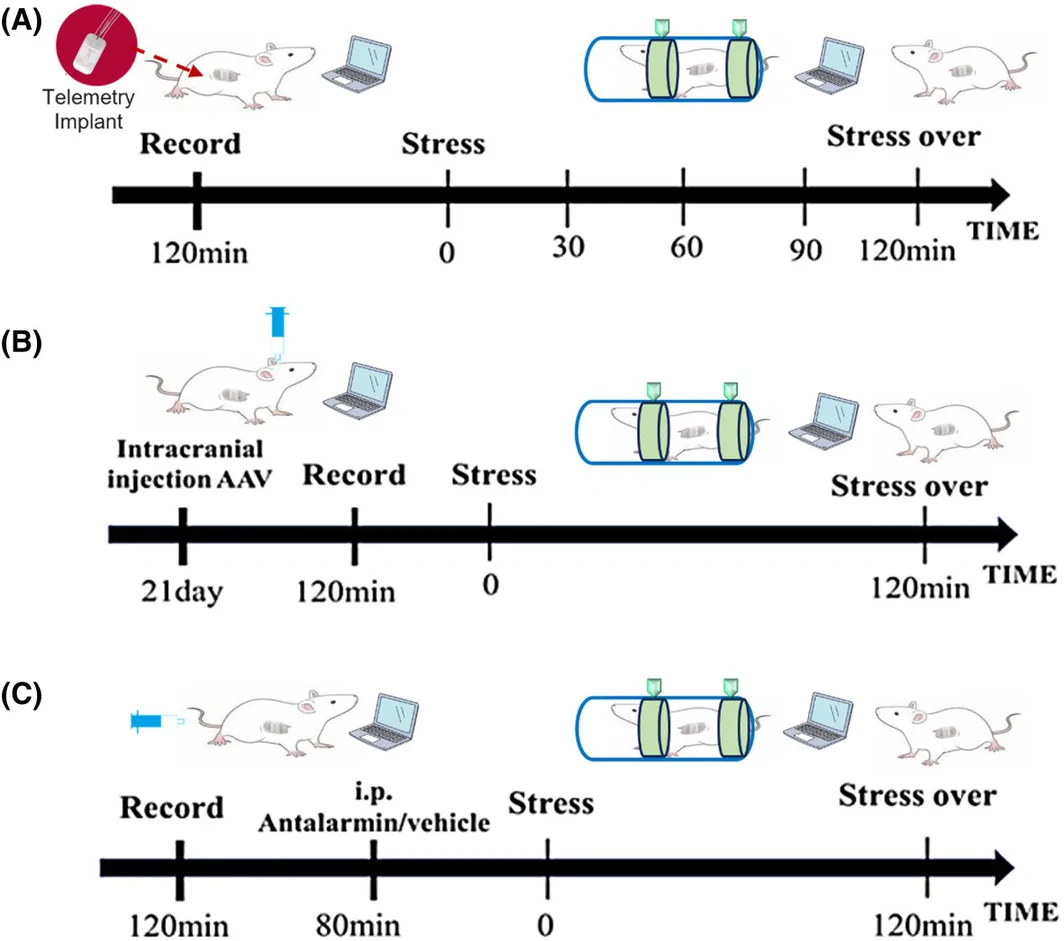
Schematic overview of experimental process. (A) Establish an acute restraint stress model; (B) Acute stress following CRISPR/Cas9‐medicated CRH knockdown in the PVN. (C) Acute stress following CRHR1 antagonist intervention. i.p., intraperitoneal injection (*n* = 10) unless otherwise indicated.

### 2.3 Restraint scheme

Rats underwent a single 2‐h restraint using a cylindrical device (6 cm diameter, 15 cm length) with ventilation holes, adjustable to the size of the animals. The procedure took place between 10:00 am and 12:00 pm to align with the circadian rhythm of CRH secretion. Prior to restraint, baseline blood pressure was recorded for 1 to 2 h using a telemetry system, with food and water available. During restraint, the animals were monitored for respiratory comfort, and both food and water were withheld. For Figure 2A, each animal's baseline value was calculated as the mean of measurements obtained during the final 30 min before restraint. For Figures 4A and 5A, baseline and restraint‐period values were calculated for each animal by averaging all recorded measurements from −120 to 0 min and from 10 to 120 min, respectively.

**FIGURE 2:**
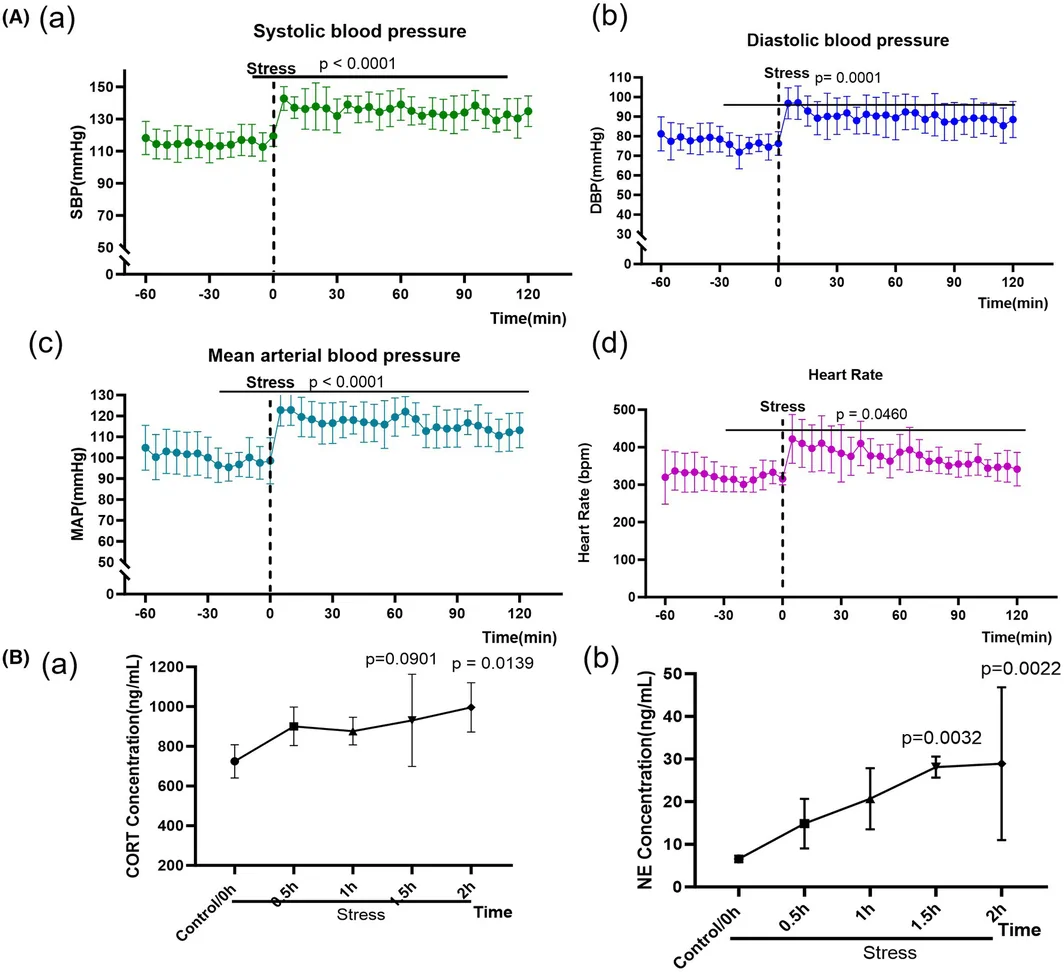
Acute restraint stress increases blood pressure, heart rate, and circulating stress hormones. (A) Continuous recordings of systolic blood pressure (SBP; a), diastolic blood pressure (DBP; b), mean arterial pressure (MAP; c), and heart rate (HR; d) before and during restraint (SBP, DBP, and MAP: *N* = 10; HR: *N* = 5). Time courses were analyzed separately by one‐way repeated‐measures ANOVA with the Geisser–Greenhouse correction. Graph labels show two‐sided paired *t*‐tests comparing each rat's final 30‐min baseline mean with its mean during restraint (5–120 min). “Stress” marks the restraint period. (B) Serum corticosterone (CORT; a) and norepinephrine (NE; b) measured in the same rats at 0, 0.5, 1, 1.5, and 2 h (*n* = 6; 0 h, pre‐restraint). Each analyte was analyzed using the same ANOVA and Dunnett's comparisons with the matched 0‐h value without assuming sphericity. Graph labels show Dunnett‐adjusted *p* values. Data are mean ± SD.

### 2.4 Telemetry transmitter implantation and blood pressure measurements

The arterial blood pressure (ABP) of conscious rats was continuously monitored by a telemetry system (Millar, Houston, TX, USA) in free‐moving rats.

### 2.5 CRH knockdown via CRISPR/Cas9 in the PVN of rats

The recombinant AAV9 vectors used in this study were commercially prepared and purchased from Brain Case (Shenzhen, China). The CRH‐targeting vector was rAAV9‐U6‐sasgRNA(rCrf)‐CMV‐NLS‐SaCas9 (Cat. No. BC‐0614; titer: [1.0 × 10^12^] VG/mL), in which the U6 promoter drives expression of the rat Crf‐targeting SaCas9 guide RNA and the CMV promoter drives expression of nuclear‐localized SaCas9. The corresponding control vector was rAAV9‐U6‐CMV‐NLS‐SaCas9 (Cat. No. BC‐0613; titer: [1.0 × 10^12^] VG/mL), which lacked the Crf‐targeting sasgRNA cassette. Both vectors were supplied as ready‐to‐use recombinant AAV9 viral preparations. The CRH‐targeting and control AAV9 vectors were bilaterally injected into the PVN of rats in the KD and CON groups, respectively (Ma et al., 2023). The CRH‐targeting and control AAV9 vectors were bilaterally injected into the PVN of rats in the KD and CON groups, respectively. Anesthesia was induced using 3% isoflurane and maintained with 2% isoflurane. The rat's head was positioned in a stereotaxic apparatus (Shenzhen Reward Life Technology, China), and the skull was exposed along the sagittal suture. A glass electrode tip was guided to the PVN, with coordinates determined from the Paxinos and Watson rat brain atlas (AP: −1.8 mm, ML: 0.5 mm, DV: 7.8 mm). Each PVN received an injection of 50 nL of the viral vector. To prevent infection, ampicillin sodium was administered intramuscularly at a dose of 200 mg/kg twice daily for 1 week. The peak virus expression was assessed 21 days post‐injection, after which the acute stress experiment commenced.

### 2.6 CRHR1 and corticoid inhibition protocol

Antalarmin hydrochloride, a selective CRHR1 antagonist (Briscoe et al., 2000), was purchased from MedChemExpress (MCE; Cat. No. HY‐103377). Antalarmin hydrochloride was prepared at a concentration of 2 mg/mL in a vehicle containing 10% DMSO, 40% PEG300, 5% Tween‐80, and 45% sterile saline, and was administered intraperitoneally at 20 mg/kg 80 min before restraint stress. Mifepristone, a glucocorticoid receptor antagonist, was purchased from AmBeed (Cat. No. A423786). Mifepristone was prepared at a concentration of 10 mg/mL in 0.9% saline containing 5% DMSO and Tween 20 and was administered intraperitoneally at 20 mg/kg/day for 14 consecutive days, with an injection volume of approximately 2 mL/kg (Lin et al., 2022). Vehicle‐control rats received an equivalent volume of the corresponding vehicle. Blood pressure measurements were recorded after drug or vehicle administration.

### 2.7 Western blotting

After stress exposure, SD rats were anesthetized with 3% isoflurane and euthanized by decapitation, after which the PVN, LC, and NTS were rapidly collected and frozen. Soluble proteins were separated by 12.5% SDS‐PAGE (Shanghai Epizyme Biomedical Technology, China) and transferred to polyvinylidene fluoride (PVDF; Merck Millipore; Cat. No. ISEQ00010) membranes. Membranes were blocked with Tris‐buffered saline containing 0.1% Tween‐20 and 5% non‐fat milk. They were incubated overnight at 4°C with a primary CRH antibody (CRH Rabbit pAb A1122, 1:1600, ABclonal, USA). This antibody has been validated by the manufacturer for western blot and ELISA on human, mouse and rat samples, as documented in the official product datasheet. Subsequent results demonstrated that PVN was significant differences in detection indicators between the group that knocked out crh and the control group, indicating that the knockout of crh met the expected experimental hypothesis and was an effective knockout. After washing, membranes were treated with diluted secondary antibodies (Goat anti‐Rabbit IgG, 1:16000, AB0101, Abways, China) for 1 h at room temperature. Chemiluminescent signals were developed using an enhanced chemiluminescence (ECL) reagent (ShareBio; Cat. No. SB‐WB004) and detected using an imaging system.

### 2.8 Enzyme‐ linked immunosorbent assay

Blood of rats was collected via the tail vein, and serum was centrifuged for later analysis. Serum NE and CORT concentrations were measured using commercially available ELISA kits for norepinephrine (NE; Elabscience; Cat. No. E‐EL‐0047) and corticosterone (CORT;Jiangsu Boshen Biotechnology Co., Ltd., China; Cat. No. BS‐E11437R2), respectively, according to the manufacturer's instructions.

### 2.9 Statistical analysis

Data are presented as mean ± SD. Analyses were performed using GraphPad Prism. Normality was assessed using the Shapiro–Wilk test. Figure 2 time courses were analyzed separately for each outcome using one‐way repeated‐measures ANOVA with the Geisser–Greenhouse correction. In Figure 2A, each rat's mean baseline value during the final 30 min before restraint was compared with its mean during restraint (5–120 min) using a two‐sided paired *t*‐test. In Figure 2B, each of the four subsequent time points was compared with the matched 0‐h value using Dunnett's multiple‐comparisons test without assuming sphericity. Figures 4 and 5 were analyzed using two‐way repeated‐measures ANOVA with group/treatment and stress condition as factors, followed by Šídák's multiple‐comparisons test. Statistical significance was defined as *p* < 0.05. Sample sizes were based on prior experience and experimental requirements, without an a priori power calculation, and are reported in the figure legends.

## 3 RESULTS

### 3.1 Restraint stress induces sustained increases in blood pressure and sympathetic‐neuroendocrine activation

Acute restraint elicited an immediate elevation in cardiovascular parameters (Figure 2A). Repeated‐measures one‐way ANOVA with the Geisser–Greenhouse correction showed significant overall effects of time: SBP *F*(5.322, 47.902) = 14.65, *p* = 6.017 × 10^−9^; DBP *F*(4.266, 38.393) = 9.604, *p* = 1.250 × 10^−5^; MAP *F*(3.807, 34.264) = 13.32, *p* = 1.605 × 10^−6^; HR *F*(2.791, 11.163) = 4.206, *p* = 0.0342. For the baseline‐to‐restraint comparisons, the final 30‐min baseline and the restraint period were averaged separately for each rat, and the resulting two values were compared using a two‐sided paired *t*‐test. SBP increased from 115.53 ± 1.88 to 135.20 ± 2.17 mmHg (*p* = 8.119 × 10^−5^); DBP increased from 75.01 ± 1.34 to 90.24 ± 2.20 mmHg (*p* = 8.425 × 10^−5^); MAP increased from 97.49 ± 1.95 to 116.64 ± 2.05 mmHg (*p* = 7.051 × 10^−5^); HR increased from 316.95 ± 10.09 to 375.55 ± 17.09 bpm (*p* = 0.0145). These period averages are presented as mean ± SD. The time courses showed an initial peak followed by a decline during continued restraint.

To assess activation of the stress neuroendocrine system, we measured circulating CORT and NE levels in serial blood samples collected via the tail vein. Repeated‐measures one‐way ANOVA with the Geisser–Greenhouse correction did not show a significant omnibus effect of time on CORT (*F*(1.620, 8.098) = 3.440, *p* = 0.0888), whereas the omnibus time effect on NE was significant (*F*(1.279, 6.397) = 6.374, *p* = 0.0379). Dunnett‐adjusted comparisons with the same animal's 0‐h value included all four subsequent time points. CORT was higher than the 0‐h value at 0.5 h (*p* = 0.0480), 1 h (*p* = 0.0486), and 2 h (*p* = 0.0114), but not at 1.5 h (*p* = 0.2885). NE was higher at 0.5 h (*p* = 0.0407), 1 h (*p* = 0.0128), and 1.5 h (*p* = 3.470 × 10^−6^), but the comparison at 2 h did not reach statistical significance (*p* = 0.0859). These findings show sustained cardiovascular responses during restraint together with time‐specific changes in circulating stress hormones.

### 3.2 Knockdown CRH in the PVN attenuated acute stress‐induced increases in blood pressure and CORT and NE levels

To determine whether PVN‐derived CRH is required for restraint‐induced increases in blood pressure, CRH expression was selectively reduced using CRISPR/Cas9‐mediated knockdown (KD) through bilateral microinjection of rAAV9‐U6‐sasgRNA(rCrf)‐CMV‐NLS‐SaCas9‐3xHA‐WPRE‐BGHpA vector into the PVN (Figure 3A,B). Twenty‐one days after AAV9 injection, PVN‐CRH levels were significantly reduced compared with control (CON) rats (*p* = 0.0187) without affecting CRH levels in the LC or NTS (Figure 3C), confirming a region‐specific CRH knockdown. The overall knockdown main effect was not significant for SBP, DBP, MAP, or HR. Two‐way repeated‐measures ANOVA showed significant stress and knockdown × stress interaction effects for all four cardiovascular outcomes. Restraint stress significantly increased SBP, DBP, MAP, and HR in CON rats. In KD rats, DBP and MAP increased significantly, whereas the SBP and HR changes did not reach statistical significance. In KD rats, SBP changed from 140.91 ± 3.54 to 149.56 ± 6.83 mmHg (*p* = 0.0509), DBP from 85.87 ± 2.50 to 91.32 ± 4.16 mmHg (*p* = 0.0312), and MAP from 109.90 ± 3.07 to 117.18 ± 5.19 mmHg (*p* = 0.0168). In control rats, SBP changed from 122.52 ± 1.84 to 152.50 ± 3.60 mmHg (*p* = 7.738 × 10^−6^), DBP from 76.91 ± 2.58 to 99.73 ± 1.54 mmHg (*p* = 5.192 × 10^−7^), and MAP from 101.58 ± 2.10 to 125.41 ± 2.43 mmHg (*p* = 1.704 × 10^−6^) (Figure 4A). HR increased from 318.70 ± 8.32 to 417.97 ± 32.05 bpm in control rats (*p* = 6.528 × 10^−6^), whereas KD rats showed a smaller change from 371.52 ± 27.86 to 394.81 ± 18.78 bpm (*p* = 0.1077) (Figure 4A). In addition, stress‐period CORT and NE levels were lower in KD rats than in controls (CORT: *p* = 0.0051; NE: *p* = 0.0070) (Figure 4B). These results support an important role for PVN CRH in acute stress‐induced sympathetic and cardiovascular activation.

**FIGURE 3:**
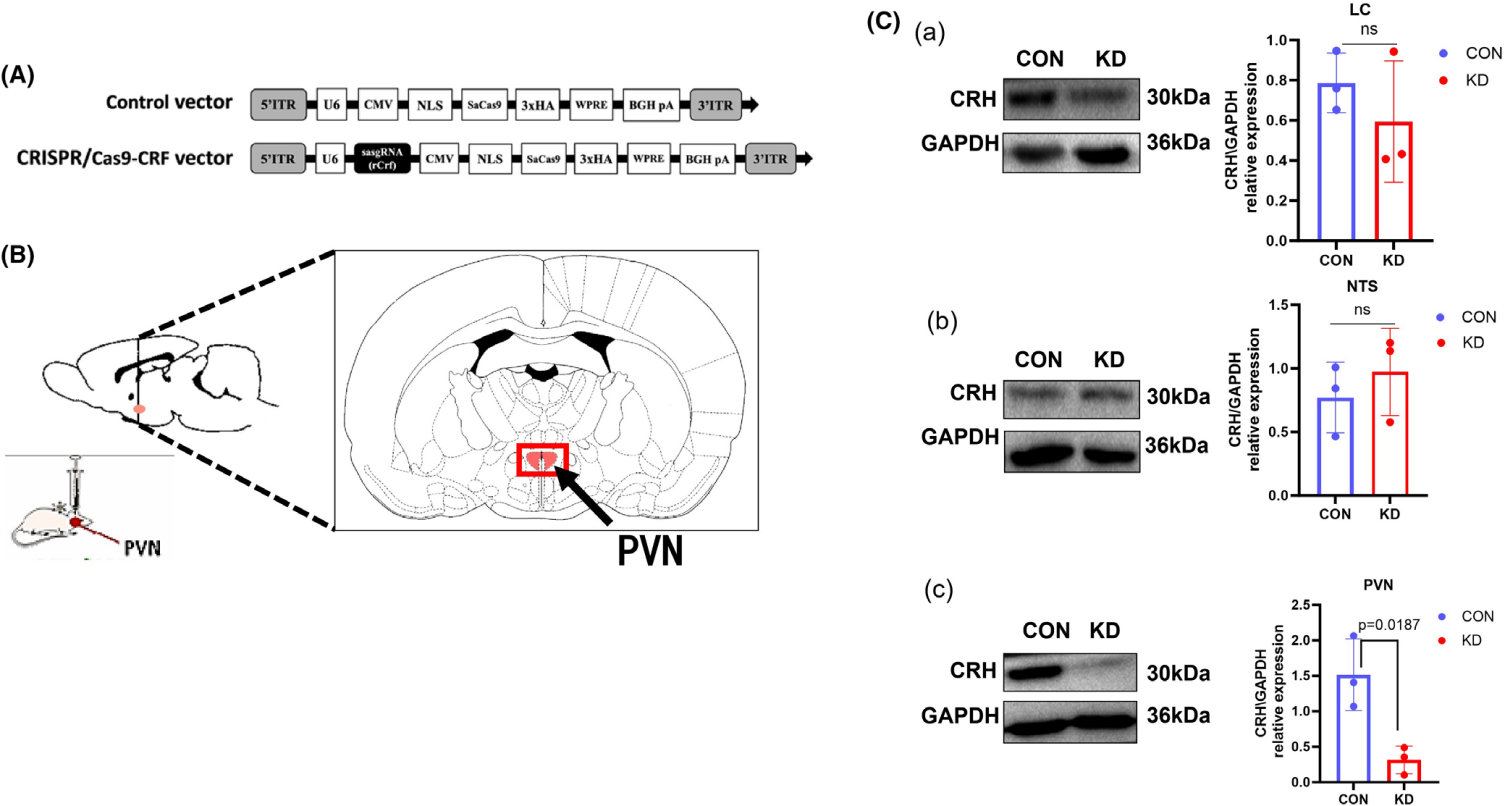
CRH knockdown selectively reduced CRH protein expression in the PVN. (A) Diagram of the rAAV9‐U6‐sasgRNA(rCrf)‐CMV‐NLS‐SaCas9‐3xHA‐WPRE‐BGHpA knockdown vector and control construct. (B) Schematic of bilateral PVN microinjection. (C) CRH protein levels in the LC (a), NTS (b), and PVN (c) at 21 days post‐injection (*n* = 3). Comparisons between the CON and KD groups were performed using unpaired *t*‐tests. Exact *p* values for significant comparisons are shown directly on the graphs; ns indicates no significant difference. Data are expressed as mean ± SD. Full‐length Western blot images with labeled molecular weight marker positions are provided in Supplementary Figure S1.

**FIGURE 4:**
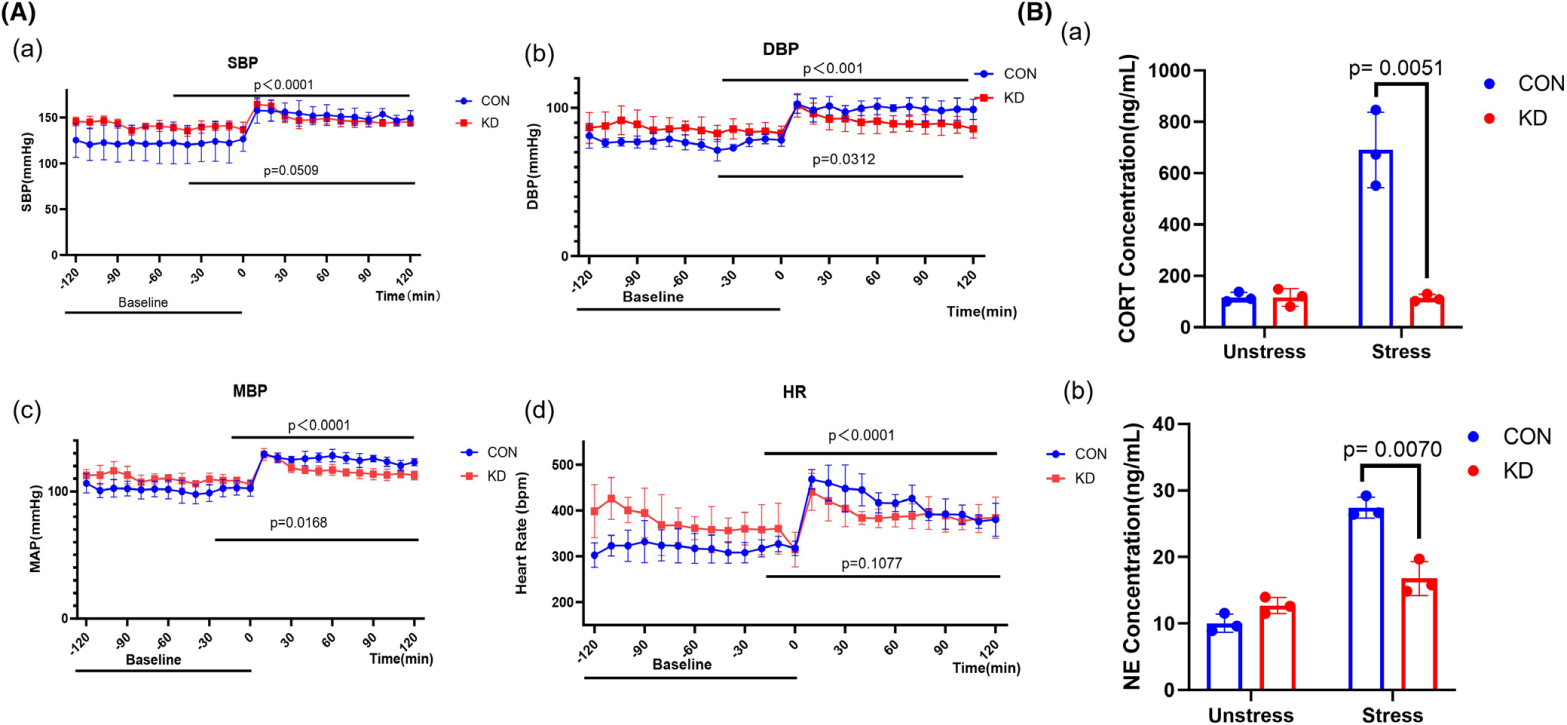
CRH knockdown attenuated acute stress‐induced cardiovascular and hormonal responses. (A) Cardiovascular responses to restraint stress in CRH knockdown (KD) and control (CON) rats (*n* = 6): systolic blood pressure (SBP; a), diastolic blood pressure (DBP; b), mean arterial pressure (MAP; c), and heart rate (HR; d). (B) Serum corticosterone (CORT; a) and norepinephrine (NE; b) responses (*n* = 3). Baseline and stress‐period measurements were averaged for each rat. All outcomes were analyzed using two‐way repeated‐measures ANOVA with group (CON vs. KD) as the group factor and stress condition (baseline vs. stress) as the repeated factor, followed by Šídák's multiple‐comparisons test. Graph labels show Šídák‐adjusted *p* values for within‐group baseline–stress comparisons in panel A and CON–KD comparisons during stress in panel B. Data are mean ± SD.

Two‐way repeated‐measures ANOVA results are reported below as knockdown effect, stress effect, and knockdown × stress interaction, respectively. For cardiovascular outcomes, all effects had *F*(1, 10): SBP, *F* = 1.768, *p* = 0.2131; *F* = 68.25, *p* = 8.875 × 10^−6^; *F* = 20.81, *p* = 0.0010. DBP, *F* = 0.0101, *p* = 0.9219; *F* = 113.3, *p* = 8.935 × 10^−7^; *F* = 42.77, *p* = 6.559 × 10^−5^. MAP, *F* = 0.0009, *p* = 0.9764; *F* = 97.56, *p* = 1.780 × 10^−6^; *F* = 27.62, *p* = 0.0004. HR, *F* = 2.038, *p* = 0.1839; *F* = 65.07, *p* = 1.095 × 10^−5^; *F* = 25.01, *p* = 0.0005. For hormonal outcomes, all effects had *F*(1, 4): CORT, *F* = 50.33, *p* = 0.0021; *F* = 35.85, *p* = 0.0039; *F* = 36.59, *p* = 0.0038. NE, *F* = 16.44, *p* = 0.0154; *F* = 106.4, *p* = 0.0005; *F* = 40.98, *p* = 0.0031.

### 3.3 CRHR1 antagonism attenuated acute stress‐induced HR, CORT, and NE responses

To determine the role of CRHR1 in mediating the acute stress response, we assessed the effect of the CRHR1 antagonist antalarmin on stress‐induced increases in blood pressure, CORT, and NE levels in rats. The overall treatment main effect was not significant for SBP, DBP, MAP, or HR. During restraint, SBP changed from 147.04 ± 7.64 to 153.31 ± 2.22 mmHg (*p* = 0.2489), MAP from 115.69 ± 6.77 to 119.16 ± 2.30 mmHg (*p* = 0.5703), and HR from 354.12 ± 21.36 to 367.06 ± 10.91 bpm (*p* = 0.1969) in antalarmin‐treated rats. In vehicle‐treated rats, SBP increased significantly from 141.32 ± 8.86 to 156.73 ± 5.70 mmHg (*p* = 0.0068), and DBP from 89.82 ± 4.61 to 99.59 ± 4.23 mmHg (*p* = 0.0123). MAP increased numerically from 116.03 ± 5.53 to 124.58 ± 4.92 mmHg without reaching statistical significance (*p* = 0.0750), whereas HR increased significantly from 324.20 ± 15.67 to 406.43 ± 38.07 bpm (*p* = 5.356 × 10^−6^) (Figure 5A). No significant baseline‐to‐stress increase in DBP was detected in antalarmin‐treated rats (*p* = 0.6420). Stress‐period CORT and NE levels were lower after antalarmin than after vehicle (CORT: *p* = 0.0004; NE: *p* = 0.0158) (Figure 5B). Treatment × stress interactions were significant for HR (*p* = 0.0001), CORT (*p* = 0.0002), and NE (*p* = 0.0039), but not for SBP (*p* = 0.1237), DBP (*p* = 0.0829), or MAP (*p* = 0.3279). These findings support CRHR1 involvement in acute stress‐induced HR and hormonal responses, but do not establish significant attenuation of the blood pressure response by antalarmin.

**FIGURE 5:**
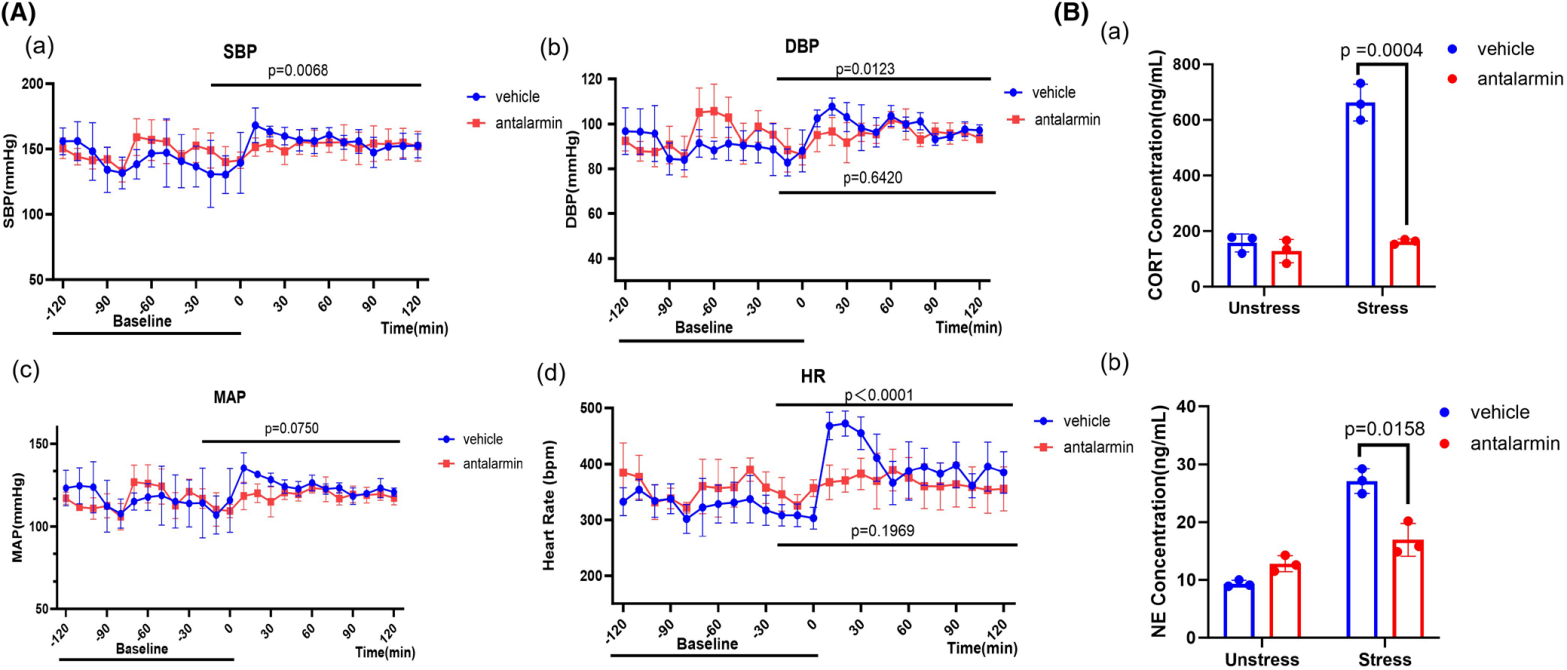
CRHR1 antagonism attenuated acute stress‐induced HR and hormonal responses. (A) Effects of intraperitoneal antalarmin or vehicle treatment on systolic blood pressure (SBP; a), diastolic blood pressure (DBP; b), mean arterial pressure (MAP; c), and heart rate (HR; d) during restraint stress (*n* = 5). (B) Serum corticosterone (CORT; a) and norepinephrine (NE; b) before and after restraint. Baseline and stress‐period measurements were averaged for each rat. All outcomes were analyzed using two‐way repeated‐measures ANOVA with treatment (vehicle vs. antalarmin) as the group factor and stress condition (baseline vs. stress) as the repeated factor, followed by Šídák's multiple‐comparisons test. Graph labels show Šídák‐adjusted *p* values for within‐group baseline–stress comparisons in panel A and vehicle–antalarmin comparisons under stress in panel B. Data are mean ± SD.

Two‐way repeated‐measures ANOVA results are reported below as treatment effect, stress effect, and treatment × stress interaction, respectively. For cardiovascular outcomes, all effects had *F*(1, 8): SBP, *F* = 0.0400, *p* = 0.8464; *F* = 16.67, *p* = 0.0035; *F* = 2.959, *p* = 0.1237. DBP, *F* = 0.0104, *p* = 0.9214; *F* = 10.46, *p* = 0.0120; *F* = 3.925, *p* = 0.0829. MAP, *F* = 0.9829, *p* = 0.3505; *F* = 6.065, *p* = 0.0392; *F* = 1.086, *p* = 0.3279. HR, *F* = 0.1619, *p* = 0.6979; *F* = 90.98, *p* = 1.207 × 10^−5^; F = 48.21, *p* = 0.0001. For hormonal outcomes, all effects had *F*(1, 4): CORT, *F* = 82.70, *p* = 0.0008; *F* = 208.9, *p* = 0.0001; *F* = 159.5, *p* = 0.0002. NE, *F* = 9.223, *p* = 0.0385; *F* = 93.72, *p* = 0.0006; *F* = 36.13, *p* = 0.0039.

### 3.4 Glucocorticoid receptor blockade did not significantly alter serum NE levels

To evaluate whether NE elevation during acute stress depends on glucocorticoid signaling, rats were treated with the glucocorticoid receptor antagonist mifepristone. Mifepristone produced a transient rise in SBP on day 5 (106.26 ± 4.11 mmHg to 125.68 ± 4.30 mmHg; *p* = 0.0003), followed by lower SBP than in controls on day 14 (97.64 ± 1.68 mmHg in mifepristone‐treated rats; *p* = 0.0025; Figure 6A). ELISA analysis of serum samples (three biological replicates per group, measured in eight technical replicates) showed the serum CORT levels were significantly decreased in mifepristone‐treated rats (*p* = 0.0017, Figure 6B), whereas no significant difference in NE was detected between mifepristone‐treated and control rats (*p* > 0.05; Figure 6C). This finding does not establish complete independence of circulating NE from glucocorticoid signaling.

**FIGURE 6:**
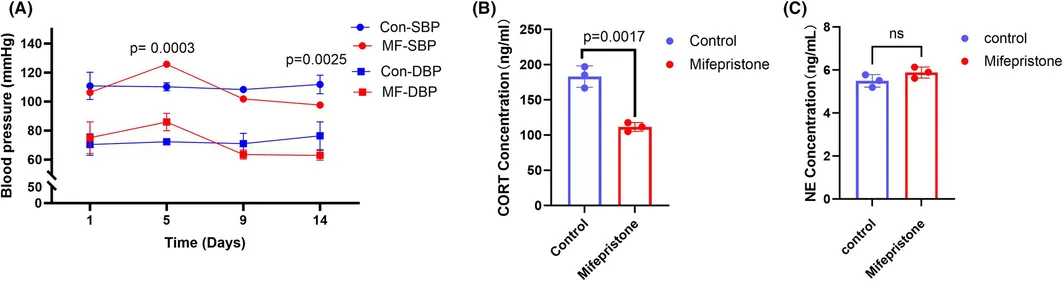
Glucocorticoid receptor blockade did not significantly alter serum NE levels. (A) Changes in blood pressure during mifepristone treatment (*n* = 5), analyzed using two‐way ANOVA. Exact *p* values for significant comparisons are shown directly on the graph. (B, C) Serum CORT (B) and NE (C) concentrations following treatment (*n* = 3 biologically independent animals per group). Comparisons between the control and mifepristone groups were performed using unpaired *t*‐tests. The exact *p* value for the significant CORT comparison is shown directly on the graph; ns indicates no significant difference in NE concentration. Data are expressed as mean ± SD. CORT, corticosterone; NE, norepinephrine.

## 4 DISCUSSION

The present study identifies corticotropin‐releasing hormone (CRH) in the PVN as an important mediator linking acute stress to sympathetic activation and blood pressure elevation. Acute restraint stress markedly increased systolic blood pressure and heart rate, with higher serum NE and CORT at selected sampling times, consistent with concurrent activation of the HPA axis and sympatho‐adrenal system (Ulrich‐Lai & Herman, 2009). These observations reinforce the concept that the PVN serves as an integrative hub in coordinating neuroendocrine and autonomic responses. We found that CRH knockdown in the PVN significantly attenuated stress‐induced increases in blood pressure and NE, suggesting that PVN‐CRH may facilitate sympathetic activation in addition to its classical endocrine role in the HPA axis (Ma et al., 2023). This dual regulatory capacity positions CRH as a potential convergence point between hormonal and autonomic stress pathways. Interestingly, inhibition of glucocorticoid signaling did not affect NE responses, suggesting that NE elevation during acute stress is not solely secondary to glucocorticoid release. These findings support partial functional independence between the HPA and SAM systems during acute stress.

The early development of hypertension is closely associated with psychological stress. Both physical and psychological stressors can elicit cardiovascular responses through coordinated autonomic and neuroendocrine stress pathways (Brotman et al., 2007; Ulrich‐Lai & Herman, 2009). In the current study, a 2‐h restraint paradigm successfully established an acute stress model characterized by sustained elevations in BP and HR and increased sympathetic activity. Comparisons with the 0‐h value showed higher serum CORT at 0.5, 1, and 2 h, although the omnibus CORT time effect was not significant; increases at selected times are consistent with previous reports (Ding et al., 2021). To avoid adaptation‐related blunting of CORT responses observed in repeated stress paradigms (Ding et al., 2021), only a single exposure was used. Moreover, anesthetic procedures can influence circulating corticosterone measurements; both pentobarbital and isoflurane alter acute stress responses in rats, and brief isoflurane or CO_2_ exposure can affect plasma corticosterone (Wu et al., 2015; Zardooz et al., 2010). These methodological considerations reduce, but do not eliminate, potential anesthetic effects on endocrine measurements. CORT, as the terminal effector of the HPA axis, is a key marker of acute stress. CRH release from the PVN triggers pituitary ACTH secretion and adrenal CORT synthesis. Accordingly, the present study does not establish the acute time course of endogenous CRH protein abundance after restraint; the mechanistic interpretation is instead based on the PVN‐specific CRH knockdown and CRHR1 antagonist experiments.

To directly test the causal role of PVN CRH in blood pressure elevation, CRISPR/Cas9‐mediated knockdown was performed. Stress‐induced increases in systolic blood pressure, mean arterial pressure (MAP), and plasma CORT were significantly blunted in CRH‐knockdown rats. This observation aligns with earlier evidence that central CRH contributes to stress‐induced pressor responses (Morimoto et al., 1993). Although CRH signaling is a key component of the stress response, its functional consequences vary with receptor subtype, brain region, and cell type, neural circuit, and prior stress exposure (Bale & Vale, 2004; Henckens et al., 2016). In the present study, pretreatment with the CRHR1 antagonist antalarmin significantly attenuated stress‐induced HR and CORT responses, consistent with previous reports (Briscoe et al., 2000; Traslaviña & Franci, 2011). Blood pressure responses were numerically smaller after antalarmin, but the treatment × stress interactions for SBP, DBP, and MAP were not significant. Together with the CRH knockdown results, these findings support the involvement of CRH–CRHR1 signaling in acute stress responses, while the evidence for attenuation of the pressor response is stronger for PVN CRH knockdown than for systemic CRHR1 antagonism. The neural mechanisms linking PVN CRH signaling to cardiovascular regulation remain incompletely understood. Previous studies have shown that PVN CRH neurons project to autonomic regulatory regions including the LC, NTS, and RVLM, which are critically involved in sympathetic activation and cardiovascular control. Therefore, modulation of these downstream circuits may represent potential pathways through which PVN CRH signaling contributes to the cardiovascular responses observed in the present study. However, the precise neural pathways and synaptic mechanisms underlying the effects of PVN CRH signaling on stress‐induced cardiovascular regulation require further investigation.

Given that antalarmin was delivered intraperitoneally, its primary site of action remains uncertain. Systemically administered antalarmin is centrally bioavailable, and therefore its effects cannot be assigned exclusively to a single CRHR1‐expressing brain region (Habib et al., 2000). At the same time, systemic CRHR1 blockade may influence both central and peripheral CRHR1‐dependent processes. Thus, the antalarmin experiment should be interpreted as complementary to, rather than independent proof of, the PVN‐specific CRH knockdown findings. Future studies incorporating optogenetic or chemogenetic interrogation of PVN CRH circuits, as well as region‐specific receptor manipulations, will be required to resolve the spatial specificity of CRH signaling in cardiovascular control.

Beyond its endocrine role within the HPA axis, PVN CRH neurons coordinate stress‐related responses, and CRH activates the locus coeruleus–norepinephrine (LC–NE) system, which contributes to autonomic adaptation (Fuzesi et al., 2016; Valentino et al., 1983, 1993). The increase in plasma NE is consistent with recruitment of the LC–NE/sympatho‐adrenal system. Importantly, no significant between‐group difference in NE was detected after glucocorticoid receptor blockade. This result is consistent with, but does not establish, partial independence from glucocorticoid signaling. These observations suggest that LC–NE/sympatho‐adrenal activation can occur partly independently of slower endocrine feedback. This functional dissociation between the HPA and SAM systems may help explain why modulation of glucocorticoid signaling does not necessarily normalize sympathetic activation, which remains an important contributor to hypertension and cardiovascular risk (Grassi, 2021; Ulrich‐Lai & Herman, 2009).

In conclusion, our data identify PVN CRH signaling as an important mediator of acute stress–induced BP elevation and NE release; however, no significant difference in serum NE was detected after glucocorticoid receptor blockade. The CRH–CRHR1 pathway integrates HPA and sympathetic responses to coordinate cardiovascular adaptation to stress (Figure 7). These findings provide mechanistic insight into the neuroendocrine regulation of stress‐induced blood pressure elevation and suggest that targeting central CRH–CRHR1 signaling may offer a promising strategy to mitigate acute stress–related sympathetic overactivation and cardiovascular risk.

**FIGURE 7:**
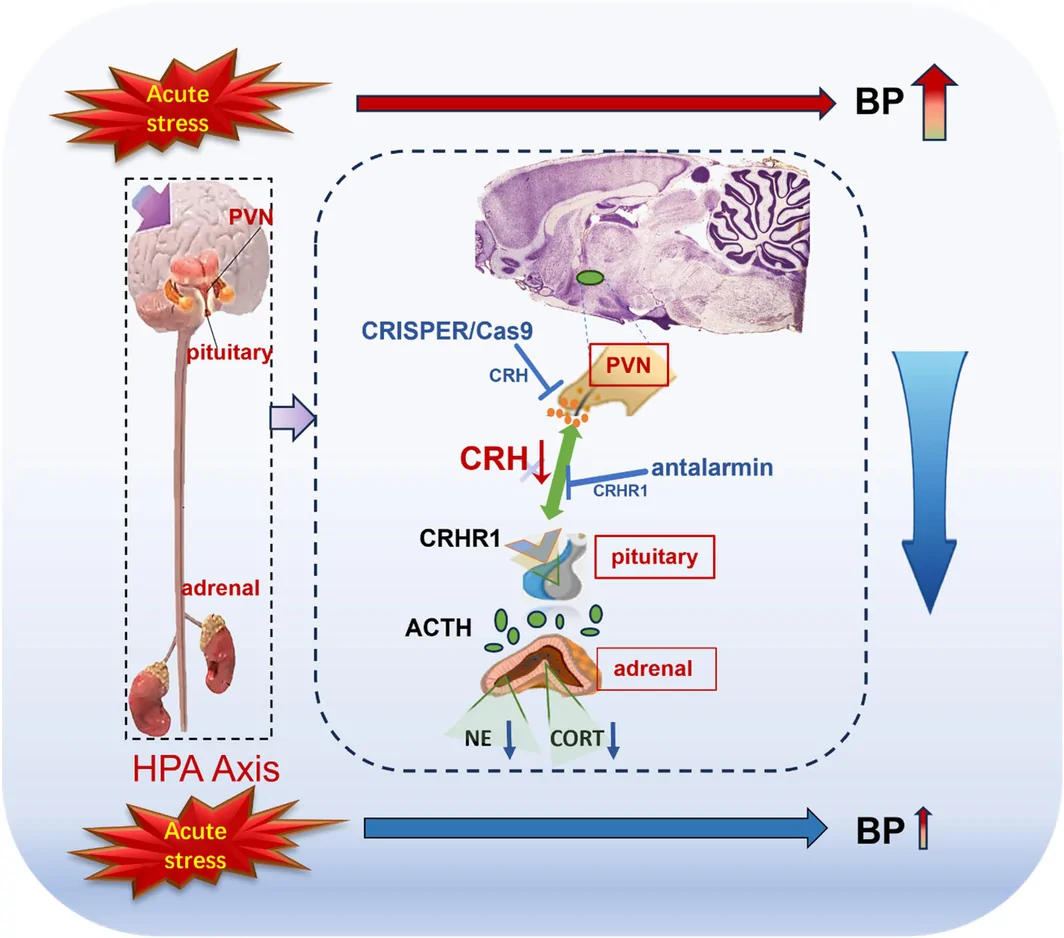
Proposed mechanism model. Schematic illustrating the role of PVN‐derived CRH in activating ACTH release, CORT secretion, LC‐NE signaling, and blood pressure regulation during acute stress. ACTH, adrenocorticotropic hormone; BP, blood pressure; CORT, corticosterone; CRH, corticotropin‐releasing hormone; NE, norepinephrine; PVN, paraventricular nucleus.

## AUTHOR CONTRIBUTIONS

L.N. was responsible for data curation, funding acquisition, and writing the original draft. T.Y. and L.S. were responsible for the operation and experimental methods of the experiment. L.R. was responsible for revising, organizing, and editing the original draft; L.J.W. is responsible for assisting in drawing; J.H.Z. was responsible for methodology and writing the original draft; W.H.J. was responsible for conceptualization, project management, and resources; N.S.Y. was responsible for reviewing the manuscript; L.J.X. was responsible for statistical analysis. All authors have read and approved the final version of the manuscript. Data authentication is not applicable.

## FUNDING INFORMATION

The Science and Technology Plan Program of Baoding (2472P011). The Hebei Central Guiding Science and Technology Development Fund (246Z7731G). Precision Medicine Cultivation Project of Hebei Natural Science Foundation (H2026201060). Science Research Project of Hebei Education Department (ZD2022060). S&T Program of Hebei (223777134D).

## CONFLICT OF INTEREST STATEMENT

The authors declare that the research was conducted in the absence of any commercial or financial relationships that could be construed as a potential conflict of interest.

## Supporting information


**Supplementary Figure S1.** Full‐length Western blot images used for the quantification shown in Figure 3. Representative full‐length blots for CRH and GAPDH in the PVN, NTS, and LC from control (CON) and CRH‐knockdown (KD) rats are shown. Molecular weight marker positions are indicated in kilodaltons (kDa).

## Data Availability

The datasets generated and/or analyzed during the current study are available from the corresponding author on reasonable request.
