# Supplementary material for: Corticotropin‐releasing hormone–mediated blood pressure elevation during acute stress in male rats
Source: Physiol Rep. 2026 Nov 13;14(21):e71172. doi: 10.14814/phy2.71172 (PMC13768595; doi:10.14814/phy2.71172)
Supplement: Supplementary file 1 [file PHY2-14-e71172-s001.pdf]

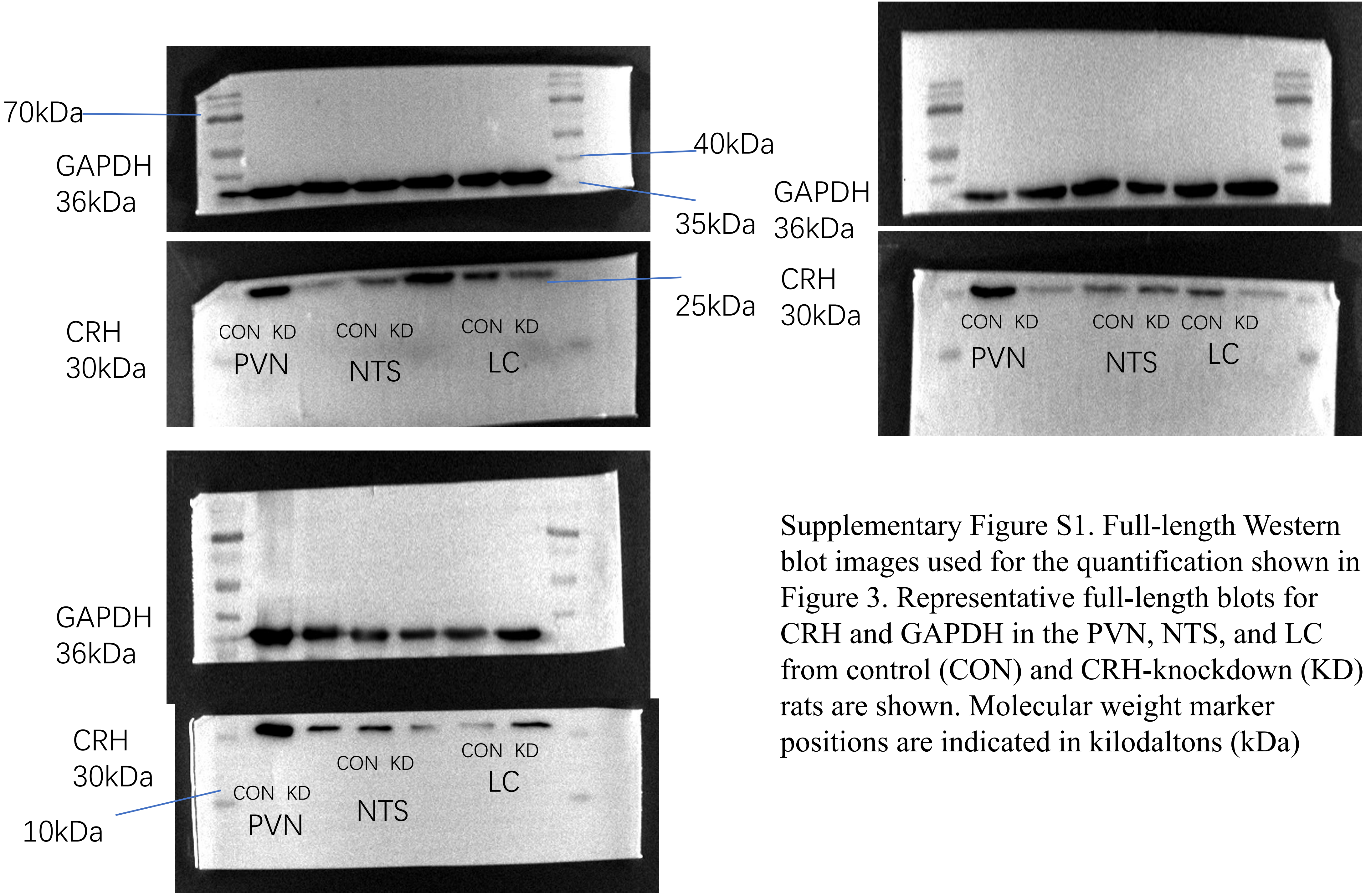

Supplementary Figure S1. Full-length Western blot images used for the quantification shown in Figure 3. Representative full-length blots for CRH and GAPDH in the PVN, NTS, and LC from control (CON) and CRH-knockdown (KD) rats are shown. Molecular weight marker positions are indicated in kilodaltons (kDa)
